# Detection and persistence of Zika virus in body fluids and associated factors: a prospective cohort study

**DOI:** 10.1038/s41598-023-48493-8

**Published:** 2023-12-06

**Authors:** Guilherme Amaral Calvet, Edna Oliveira Kara, Camila Helena Aguiar Bôtto-Menezes, Marcia da Costa Castilho, Rafael Freitas de Oliveira Franca, Ndema Habib, Armando Menezes Neto, Gerson Fernando Mendes Pereira, Silvana Pereira Giozza, Ximena Pamela Díaz Bermúdez, Tatiana Jorge Fernandes, Kayvon Modjarrad, Patrícia Brasil, Nathalie Jeanne Nicole Broutet, Ana Maria Bispo de Filippis, ZIKABRA Study Team, Morganna Costa Lima

**Affiliations:** 1 Acute Febrile Illnesses Laboratory, Evandro Chagas National Institute of Infectious Diseases, Oswaldo Cruz Foundation, Rio de Janeiro, Rio de Janeiro Brazil; 2 Department of Sexual and Reproductive Health and Research, World Health Organization, Geneva, Switzerland; 3 Department of Malaria, Tropical Medicine Foundation Doctor Heitor Vieira Dourado (FMT-HVD), Manaus, Amazonas Brazil; 4 School of Health Sciences, Amazonas State University (UEA), Manaus, Amazonas Brazil; 5 Department of Virology and Experimental Therapy, Institute Aggeu Magalhães, Oswaldo Cruz Foundation, Recife, Pernambuco Brazil; 6 Department of HIV/AIDS, Tuberculosis, Viral Hepatitis and Sexually Transmitted Infections (DATHI), Ministry of Health, Brasília, Brazil; 7 Department of Public Health, University of Brasilia, Brasília, Brazil; 8 Emerging Infectious Diseases Branch, Walter Reed Army Institute of Research, Silver Spring, MD USA; 9 Flavivirus Laboratory, Oswaldo Cruz Institute, Oswaldo Cruz Foundation, Rio de Janeiro, Rio de Janeiro Brazil

**Keywords:** Viral infection, Epidemiology

## Abstract

This study aimed to analyze the detection and duration of the Zika virus (ZIKV) in plasma, urine, saliva, sweat, rectal swabs, vaginal secretions, breast milk, and semen and to explore risk factors associated with prolonged viral persistence. A prospective cohort study of symptomatic patients and their household contacts was conducted in Brazil from July 2017 to June 2019. A total of 260 individuals (184 women and 76 men) with confirmed ZIKV infection were enrolled and followed up for 12 months. ZIKV RNA was present in all body fluid specimens and detectable for extended periods in urine, sweat, rectal swabs, and semen. The longest detection duration was found in semen, with high viral loads in the specimens. ZIKV RNA clearance was associated with several factors, including age, sex, education level, body mass index, non-purulent conjunctivitis, joint pain, and whether the participant had a history of yellow fever vaccination. The influence of each of these factors on the low or fast viral clearance varied according to the specific body fluid under investigation. Recurrent ZIKV detection events after total viral clearance were observed in the cohort. Our findings provide valuable insights into the persistence and potential recurrence of ZIKV infection, highlighting the need for continued monitoring and follow-up of individuals infected with ZIKV and for effective prevention measures to reduce the risk of transmission.

## Introduction

As of December 2021, 89 countries and territories had documented evidence of autochthonous mosquito-borne transmission of the Zika virus (ZIKV). While global cases of ZIKV disease have declined since 2017, several countries in the Americas and other endemic regions still experience transmission at low levels^1^. In addition to mosquito bites, ZIKV can also be transmitted through sexual activities^2^. The estimated risk of ZIKV transmission through unprotected sex is low^2^. In response to these findings, the World Health Organization (WHO) issued guidelines and evidence-based recommendations for preventing ZIKV sexual transmission^3^.

ZIKV infections can display a variety of clinical presentations, ranging from asymptomatic^4^ to a typical febrile disease characterized by signs of rash, non-purulent conjunctivitis, pruritus, headache, fever, and joint pain with or without periarticular edema^5^. In addition, during the more recent outbreaks in French Polynesia and Brazil, two major complications were identified as associated with ZIKV infection: Congenital Zika Syndrome that includes microcephaly and other disabilities and Guillain–Barré syndrome^6–8^.

The ZIKV ribonucleic acid (RNA) has been detected in various body fluids, including blood, saliva, urine, semen, vaginal secretions, rectal swabs, sweat, spinal fluid, amniotic fluid, and breast milk^6,9–18^. The viral clearance from these fluids can occur within days to a few weeks following the acute phase of the disease, and longer ZIKV RNA persistence has been observed in urine, saliva, and semen^12,19–21^. Although prolonged ZIKV detections are commonly reported, the mechanisms responsible for such persistence and body fluid compartmentalization have yet to be fully identified.

A better understanding of ZIKV persistence in different body fluids during the post-acute phase of the infection is of public health interest. It will contribute to elucidating the natural progression of the disease. In this study, we aimed to analyze the detection and duration of ZIKV in different body fluids, explore potential risk factors associated with prolonged viral persistence and assess the possibility of recurrent ZIKV infection within 1 year of the initial acute infection.

## Results

### Baseline study participant characteristics

Between July 2017 and June 2019, 786 individuals underwent screening in Brazil, of which 260 participants were enrolled (184 women and 76 men) (Supplementary Fig. 1). Among these, 223 participants (85.8%) completed all the study visits and corresponding specimen collection. None of the participants were found to have a co-infection with either dengue (DENV) or chikungunya (CHIKV) viruses. The baseline characteristics at the time of enrollment are summarized in Table 1 and were published in more detail previously^22^.

**Table 1:** Baseline characteristics of the participants enrolled in the study.

Characteristics	Male (n = 76)	Female (n = 184)
Age, n (%)		
≤ 35	39/76 (51.3)	87/184 (47.3)
> 35	37/76 (48.7)	97/184 (52.7)
Median (IQR)	35.0 (25.5, 43.0)	36.5 (28.0, 47.0)
Education, n (%)		
Primary or lower	10/76 (13.2)	21/184 (11.4)
Secondary	50/76 (65.8)	104/184 (56.5)
University or postgraduate	16/76 (21.1)	59/184 (32.1)
Marital status, n (%)		
Single	32/76 (42.0)	86/184 (46.7)
Married	22/76 (29.0)	59/184 (32.1)
Long term relationship	19/76 (25.0)	22/184 (12.0)
Separated/divorced	3/76 (4.0)	14/184 (7.6)
Widowed	0/76 (0.0)	3/184 (1.6)
Living with other people, n (%)		
Yes	63/64 (98.4)	154/162 (95.1)
Missing	12	22
Household members, excluding self		
1–2	27/63 (42.9)	79/154 (51.3)
3 or more	36/63 (57.1)	75/154 (48.7)
Median (IQR)	3 (2,4)	2 (1,4)
Missing	13	30
Weight (kg)		
Median (IQR)	81.7 (80.0, 95.8)	67.2 (58.4, 75.3)
Height (m)		
Median (IQR)	1.71 (1.66, 1.74)	1.58 (1.55, 1.63)
Body mass index, n (%)		
< 18.50: underweight	0/76 (0.0)	3/140 (1.7)
18.50–24.99: normal weight	18/76 (23.7)	62/180 (34.4)
≥ 25.00: overweight or obese	58/76 (76.3)	115/180 (63.9)
Missing	–	4
Fever, n (%)		
On the enrollment visit or past 30 days, Yes	70/76 (92.1)	148/184 (80.4)
Skin rash, n (%)		
On the enrollment visit or past 30 days, Yes	74/76 (97.4)	183/184 (99.5)
Skin itching (pruritus), n (%)		
On the enrollment visit or past 30 days, Yes	70/76 (92.1)	175/184 (95.1)
Non-purulent conjunctivitis, n (%)		
On the enrollment visit or past 30 days, Yes	57/76 (75.0)	133/184 (72.3)
Joint pain (arthralgia), n (%)		
On the enrollment visit or past 30 days, Yes	55/76 (72.4)	157/184 (85.3)
Periarticular edema, n (%)		
On the enrollment visit or past 30 days, Yes	35/76 (46.1)	145/184 (78.8)
Received vaccine against yellow fever, n (%)		
Yes	49 (65.3)	130 (71.0)
No	5 (6.7)	15 (8.2)
Do not know	21 (28.0)	38 (20.8)
Missing	1	1

*IQR* interquartile range.

### ZIKV rRT-PCR detection

During the study period, 25,049 specimens were collected from 260 participants (Supplementary Table 1). Enrollment required participants to have a positive ZIKV rRT-PCR result in blood and/or urine specimens. For index cases, ZIKV RNA detection was performed within 48 h of the initial visit screening (V0) and a maximum of 7 days after the onset of symptoms.

#### Plasma

During the initial screening visit (V0), 27 out of 76 men (35.5%) and 63 out of 184 women (34.2%) tested positive for ZIKV RNA in plasma. Among the men who tested positive, 24 (31.6%) showed ZIKV RNA presence on at least one subsequent visit and seven (9.2%) on multiple visits. Among women, 63 (34.2%) showed detectable ZIKV RNA in plasma on at least one additional visit, while only 4 (2.2%) showed detection on two or more visits.

#### Urine

A total of 67 (82.2%) men and 157 (85.3%) women tested positive for ZIKV in urine specimens during the screening visit, showing a much higher positivity proportion than plasma. Of the 76 male participants, 74 (97.4%) had detectable viral RNA in at least one additional visit, and 64 (84.2%) had detectable viral RNA in two or more follow-up visits. In women, 172 (93.5%) had detectable ZIKV RNA in at least one additional visit, and 143 (77.7%) had detectable RNA in two or more subsequent visits.

#### Saliva

Of the 76 male participants, 53 (69.7%) had detectable ZIKV RNA in saliva specimens and 28 (36.8%) in at least one additional visit. Among women, 118 (64.1%) had detectable ZIKV RNA, and 44 (23.9%) had it in at least one additional visit.

The median time to the first ZIKV detection in saliva was similar between male and female participants [males: median 6.0 days (95% CI 5.0–7.0 days), females: median 5.5 days (95% CI 5.0–6.0 days)].

#### Sweat

The rate of ZIKV rRT-PCR positivity in sweat specimens was the lowest among all specimens. Only 27 (35.5%) male participants and 52 (32.9%) female participants had at least one detectable result during the study. Two or more positive results were rare (men: n = 5, 6.6%, and women: n = 9, 4.9%).

#### Rectal swabs

Of the 76 male participants, 43 (56.6%) tested positive on at least one visit, and among them 22 (28.9%) tested positive on at least one subsequent visit. Among the 184 female participants, 122 (66.3%) had detectable ZIKV RNA, and 60 (32.6%) tested positive in at least one additional visit.

The median time to the first ZIKV detection in rectal swabs was 9.0 days (95% CI 6.0–99.0 days) among males and 6.0 days (95% CI 5.0–7.0 days) among females.

#### Vaginal secretions

The rate of ZIKV rRT-PCR positivity in vaginal secretions specimens showed a similar profile to saliva and rectal swab specimens. Among the 184 females, 112 (60.7%) had detectable ZIKV RNA in at least one study visit, and 49 (26.6%) had further detections in at least one additional visit.

The median time to the first ZIKV detection in vaginal secretions was 6.0 days (95% CI 5.0–8.0 days).

#### Breast milk

Only six (3.3%) breastfeeding women were included in the study. Four women had a detectable ZIKV rRT-PCR result in at least one breast milk specimen.

The median time to the first ZIKV detection in breast milk was 6.5 days (95% CI 2.0 to NA days).

#### Semen

Among the 75 participants who provided semen specimens, 52 (69.3%) had detectable ZIKV rRT-PCR in at least one study visit, and 35 (46.7%) had further detections in at least one additional visit.

The median time to the first ZIKV detection in semen was 13.0 days (95% CI 9.0–16.0 days).

#### Cumulative incidence of ZIKV in different body fluids

**Figure 1:**
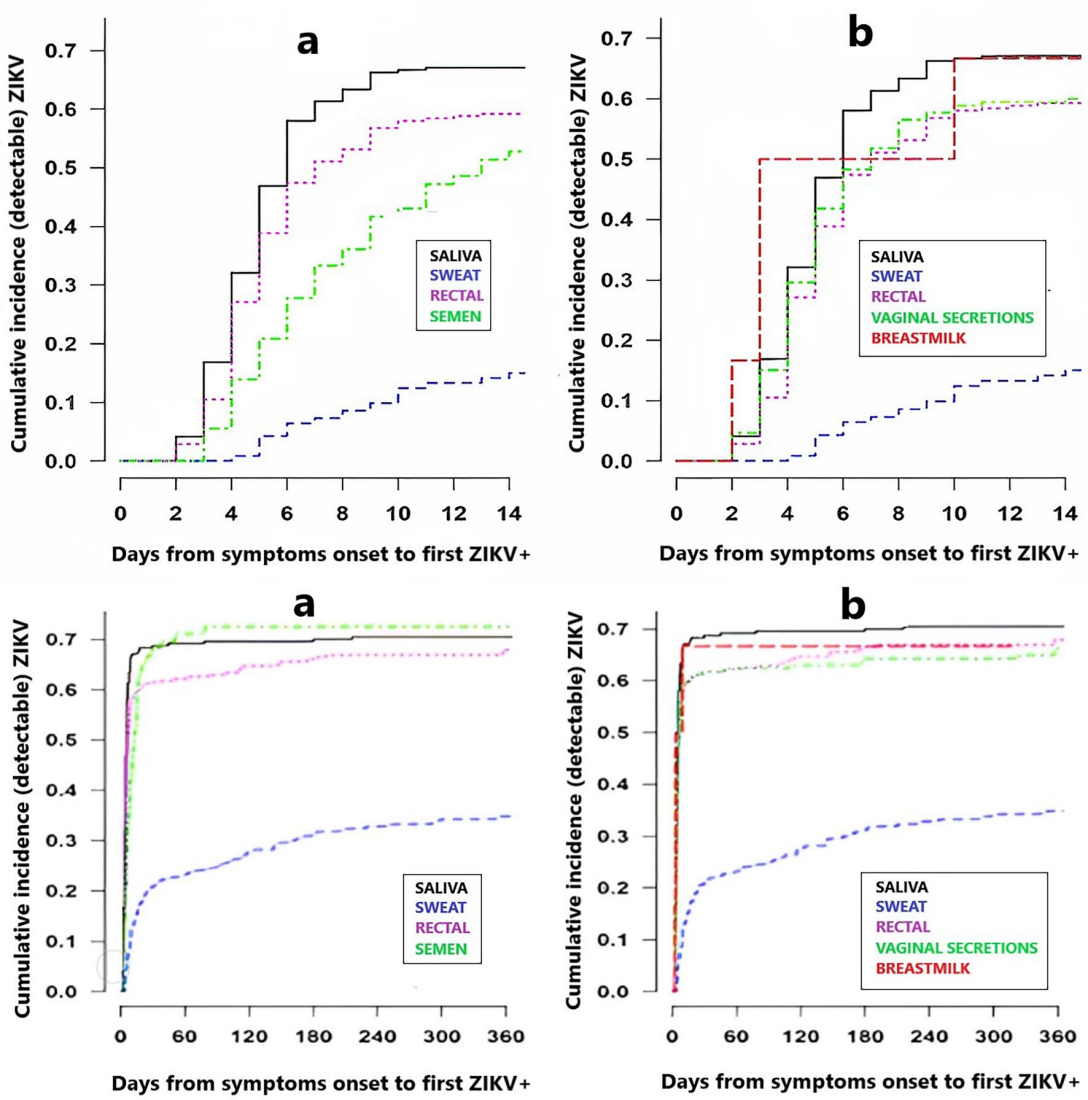
Right-censored survival-time to first ZIKV detection in body fluids following symptoms onset, (**a**) male (n = 76), (**b**) female (n = 184).

The results presented in Fig. 1a,b, and Supplementary Table 2 demonstrate the cumulative incidence of ZIKV rRT-PCR first detection across various specimen types from the onset of symptoms for male and female participants. The data indicate that by 10 days after symptom onset, more than 50% of participants had a positive detection in saliva, rectal swabs, vaginal secretions, and breast milk specimens. For sweat specimens, 18.5% (95% CI 10.9–30.2) of males and 11.5% (95% CI 7.3–17.8) of females returned a positive detection by the same period. Similarly, semen specimens showed a cumulative incidence of rRT-PCR detection of 43.7% (95% CI 33.1–56.0) of the participants by 10 days of symptom onset.

### ZIKV persistence in body fluids

#### Plasma

**Figure 2:**
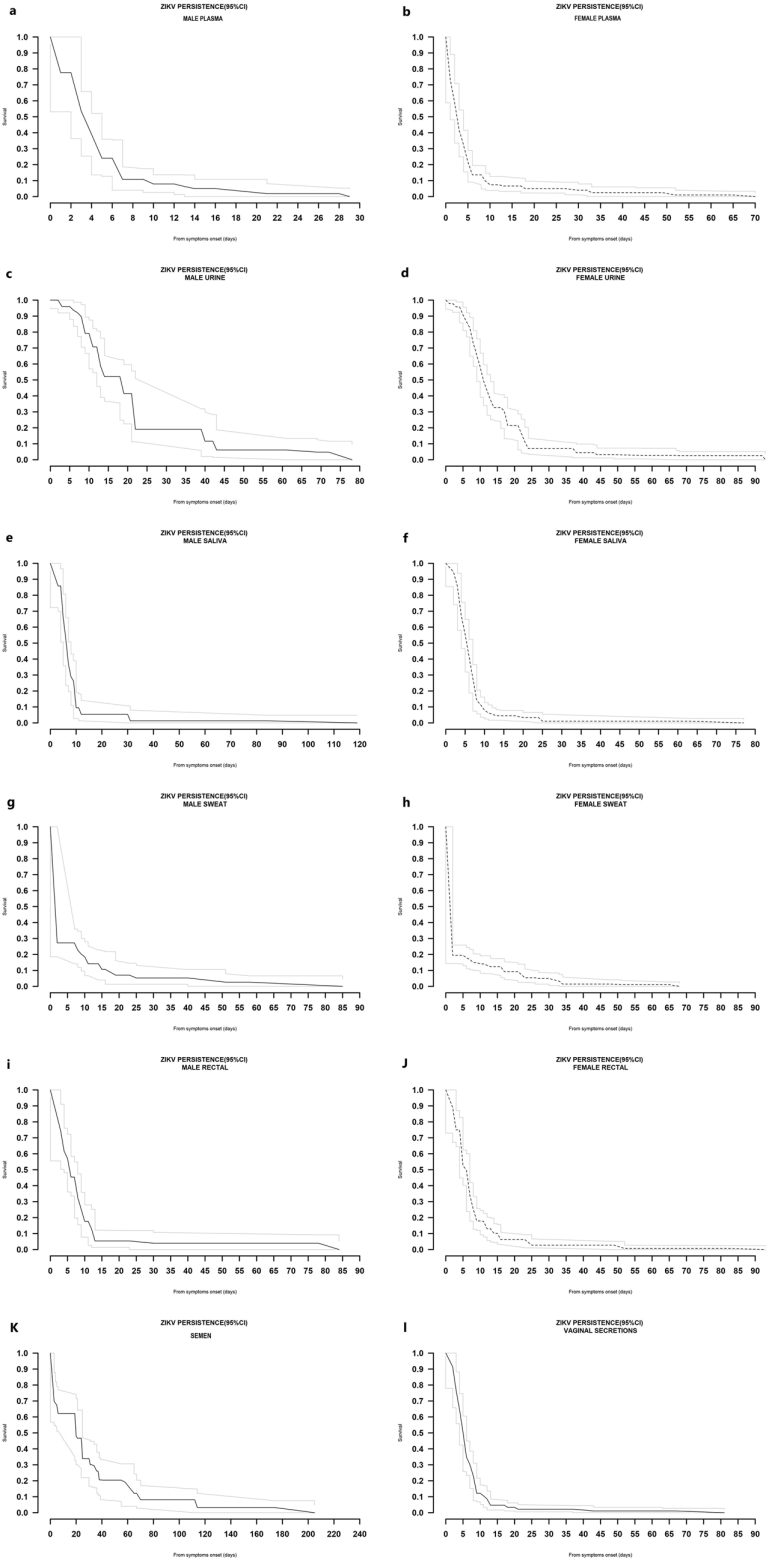
ZIKV persistence in body fluids following symptoms onset.

In men, the median ZIKV RNA persistence in plasma was 4 days (95% CI 3–5 days), with a 75th percentile of 5 days (95% CI 5–7 days) and a 95th percentile of 17 days, with 1.8% (0.2–7.8) of participants showing persistence of ZIKV RNA (Fig. 2a). Among women, the median time for virus persistence in plasma was 3 days (95% CI 2–3 days), with a 75th percentile of 5 days (95% CI 5–6 days) and a 95th percentile of 28 days. At this point, 4% (1.4–7.6) of women still had persistence of ZIKV RNA (Fig. 2b). The maximum detection of ZIKV RNA observed in plasma was 28 days for men and 65 days for women.

#### Urine

In men, the median time for viral persistence in urine was 19 days (95% CI 13–22 days), with the 75th percentile at 22 days (95% CI 22–40 days) and the 95th percentile at 62 days, where 4.8% (1.3–12.0) of participants had persistence of viral RNA (Fig. 2c). Among women, the median time for viral persistence was 11 days (95% CI 10–13 days), with the 75th percentile at 18 days (95% CI 17–22 days) and the 95th percentile at 38 days, where 4.4% (2.0–8.5) of participants still had persistence of viral RNA (Fig. 2d). The maximum observed ZIKV RNA detection in urine was 72 days for male participants and 92 days for female participants.

#### Saliva

In men, the median time for viral persistence in saliva was 7 days (95% CI 6–7 days), with the 75th percentile at 10 days (95% CI 8–10 days) and the 95th percentile at 31 days, where 1.3% (0.1–6.4) of participants still had persistence of viral RNA (Fig. 2e). In women, the median time for viral persistence in saliva was 6 days (95% CI 5–6 days), with the 75th percentile at 8 days (95% CI 7–8 days) and the 95th percentile at 13 days, where 4.5% (2.1–8.3) of participants still had persistence of ZIKV RNA (Fig. 2f). The maximum observed detection of ZIKV RNA in saliva was 84 days and 63 days for male and female participants, respectively.

#### Sweat

For male participants, the 75th percentile for ZIKV rRT-PCR persistence in sweat was 8 days (95% CI 2–11 days), while at 55 days (95th percentile), 2.6% (0.5–8.2) of the participants still had persistence of RNA (Fig. 2g). In contrast, for female participants, the 75th percentile for viral persistence was only 2 days (95% CI 2–6 days), and at 27 days (95th percentile), 4.9% (2.3–9.1) of the participants still had persistence of RNA (Fig. 2h). The maximum ZIKV detection observed in sweat was 58 days and 65 days in male and female participants, respectively.

#### Rectal swabs

Among men, the median time for viral persistence was 6 days (95% CI 4–8 days), with a 75th percentile of 10 days (95% CI 8–12 days). At the 95th percentile, 3.9% (1.1–10.1) of men still had persistence of RNA (24 days) (Fig. 2i). Among women, the median time for viral persistence was 6 days (95% CI 5–7 days), with a 75th percentile of 9 days (95% CI 8–9 days) and a 95th percentile of 24 days, where 2.8% (1.0–5.9) had RNA persistence (Fig. 2j). The maximum ZIKV detection observed in rectal swabs was 78 days and 82 days in male and female participants, respectively.

#### Vaginal secretions

The median time for viral persistence was 6 days (95% CI 5–6 days), with the 75th percentile at 8 days (95% CI 7–9 days) and the 95th percentile at 13 days, with 4.7% (2.2–8.6) still exhibiting RNA persistence (Fig. 2k). Out of the 174 vaginal specimens with detectable ZIKV RNA, only 25 (14.4%) showed simultaneous detection with blood in the same study visit. The maximum ZIKV RNA detection observed in vaginal secretions was 67 days.

#### Breast milk

The 75th percentile of time for viral persistence in breast milk was 8 days (95% CI 6–11 days), with the last detectable RNA observed 10 days after symptom onset.

#### Semen

The median time for viral persistence was 20 days (95% CI 20–25 days), with a 75th percentile of 38 days (95% CI 25–65 days) and a 95th percentile of 113 days, where 3.2% (0.7–9.2) still had persistence of RNA (Fig. 2l). The most extended ZIKV detection observed in semen was 174 days.

### ZIKV rRT-PCR cycle thresholds

Cycle threshold (Ct) values indicate the number of cycles required to amplify viral RNA to a detectable level. They are commonly used as a proxy for viral load, where the lower the Ct value, the higher the viral load. Our study observed that, among all body fluids examined, semen exhibited the lowest Ct values, particularly within the first 30 days after the onset of symptoms (Supplementary Fig. 2).

### ZIKV RNA recurrent detection

When considering any positive body fluid specimen, we identified recurrent detection of ZIKV RNA in 78 (30.0%) participants. The median time for these recurrent detections was 156 days (IQR 107.3–235.5), ranging from 78 to 357 days following symptom onset.

### Factors associated with ZIKV persistence

#### Plasma

After adjusting for joint pain, participants with post-secondary education showed a 28% (95% CI 4–46%; p = 0.023) lower ZIKV RNA clearance in plasma compared to those with either secondary or lower levels of education (Table 2).

**Table 2:** Univariate and multivariable interval censored proportional hazards model regression estimates of relative risk of being confirmed negative for ZIKV in plasma.

Selected baseline factors	Univariate model		Multivariable model	
	Univariate HR (95% CI)	Chi-sq p value	Adjusted HR (95% CI)	Chi-sq p value
Sex			–	–
Male	1.00			
Female	1.04 (0.79, 1.38)	0.78		
Age group			–	–
≤ 35	1.00			
> 35	0.93 (0.72, 1.20)	0.58		
Education level				
Secondary or lower	1.00		1.00	
Post-secondary	0.74 (0.55, 0.98)	0.03	0.72 (0.54, 0.96)	0.023
BMI category (kg/m^2^)			–	–
Normal weight (18.5–24.99)	1.00			
Overweight or obese (≥ 25.0)	0.93 (0.70, 1.23)	0.61		
Household members (excluding self)			–	–
< 3	1.00			
≥ 3	1.09 (0.82, 1.44)	0.56		
Fever today or past 30 days			–	–
(Yes vs No)	1.29 (0.91, 1.81)	0.15		
Rash today or past 30 days			–	–
(Yes vs No)	1.90 (0.61, 5.97)	0.27		
Pruritus today or past 30 days			–	–
(Yes vs No)	1.26 (0.74, 2.16)	0.39		
Non purulent conjunctivitis today or past 30 days			–	–
(Yes vs No)	1.18 (0.89, 1.57)	0.26		
Joint pain today or past 30 days				
(Yes vs No)	1.33 (0.96, 1.84)	0.09	1.38 (0.99, 1.91)	0.06
Periarticular edema today or past 30 days			–	–
(Yes vs No)	1.19 (0.91, 1.57)	0.21		
Yellow fever vaccination?			–	–
(Yes vs No)	0.99 (0.61, 1.60)	0.97		

HR > 1 implies a higher probability of being confirmed ZIKV RNA negative in plasma (lower likelihood of ZIKV persistence); HR < 1 implies a lower probability of being confirmed ZIKV RNA negative in plasma (increased likelihood of ZIKV persistence).

*ZIKV* Zika virus, *BMI* body mass index, *HR* hazard ratio, *CI* confidence interval.

#### Urine

In urine, females were 1.47 times (95% CI 1.11–1.95; p = 0.0073) more likely to be ZIKV RNA negative than their male counterparts. After adjusting for sex, older age (> 35 years) was associated with a 24% (95% CI 2–41%; p = 0.037) lower rate of ZIKV RNA clearance compared to younger age (≤ 35 years) (Table 3).

**Table 3:** Univariate and multivariable interval censored proportional hazards model regression estimates of relative risk of being confirmed negative for ZIKV in urine.

Selected baseline factors	Univariate model		Multivariable model	
	Univariate HR (95% CI)	Chi-sq p value	Adjusted HR (95% CI)	Chi-sq p value
Sex			–	–
Male	1.00		1.00	
Female	1.48 (1.12, 1.96)	0.0063	1.47 (1.11, 1.95)	0.0073
Age group				
≤ 35	1.00		1.00	
> 35	0.75 (0.58, 0.98)	0.031	0.76 (0.59, 0.98)	0.037
Education Level			–	–
Secondary or lower	1.00			
Post-secondary	0.88 (0.66, 1.16)	0.36		
BMI category (kg/m^2^)			–	–
Normal weight (18.5–24.99)	1.00			
Overweight or obese (≥ 25.0)	0.74 (0.56, 0.98)	0.038		
Household members (excluding self)			–	–
< 3	1.00			
≥ 3	0.87 (0.66, 1.15)	0.32		
Fever today or past 30 days			–	–
(Yes vs No)	0.91 (0.65, 1.29)	0.61		
Rash today or past 30 days			–	–
(Yes vs No)	1.52 (0.48, 4.86)	0.48		
Pruritus today or past 30 days			–	–
(Yes vs No)	0.97 (0.57, 1.65)	0.90		
Non purulent conjunctivitis today or past 30 days			–	–
(Yes vs No)	1.05 (0.79, 1.40)	0.74		
Joint pain today or past 30 days			–	–
(Yes vs No)	1.07 (0.77, 1.48)	0.71		
Periarticular edema today or past 30 days			–	–
(Yes vs No)	1.06 (0.81, 1.40)	0.67		
Yellow fever vaccination?			–	–
(Yes vs No)	0.83 (0.51, 1.36)	0.46		

HR > 1 implies a higher probability of being confirmed ZIKV RNA negative in urine (lower likelihood of ZIKV persistence); HR < 1 implies a lower probability of being confirmed ZIKV RNA negative in urine (increased likelihood of ZIKV persistence).

*ZIKV* Zika virus, *BMI* body mass index, *HR* hazard ratio, *CI* confidence interval.

#### Saliva

Women were 1.29 times (95% CI 0.98–1.71; p = 0.073) more likely to be ZIKV RNA negative in saliva compared to men, although with marginal statistical significance (Table 4).

**Table 4:** Univariate and multivariable interval censored proportional hazards model regression estimates of relative risk of being confirmed negative for ZIKV in saliva.

Selected baseline factors	Univariate model		Multivariable model	
	Univariate HR (95% CI)	Chi-sq p value	Adjusted HR (95% CI)	Chi-sq p value
Sex			–	–
Male	1.00		1.00	
Female	1.29 (0.98, 1.71)	0.073	1.29 (0.98, 1.71)	0.073
Age group			–	–
≤ 35	1.00			
> 35	0.93 (0.72, 1.20)	0.57		
Education level			–	–
Secondary or lower	1.00			
Post-secondary	0.88 (0.67, 1.17)	0.38		
BMI category (kg/m^2^)			–	–
Normal weight (18.5–24.99)	1.00			
Overweight or obese (≥ 25.0)	1.01 (0.77, 1.34)	0.94		
Household members (excluding self)			–	–
< 3	1.00			
≥ 3	0.88 (0.66, 1.17)	0.37		
Fever today or past 30 days			–	–
(Yes vs No)	1.06 (0.75, 1.50)	0.76		
Rash today or past 30 days			–	–
(Yes vs No)	3.0 (0.95, 9.52)	0.06		
Pruritus today or past 30 days			–	–
(Yes vs No)	1.27 (0.74, 2.20)	0.38		
Non purulent conjunctivitis today or past 30 days			–	–
(Yes vs No)	1.26 (0.94, 1.69)	0.13		
Joint pain today or past 30 days			–	–
(Yes vs No)	1.25 (0.90, 1.73)	0.19		
Periarticular edema today or past 30 days			–	–
(Yes vs No)	1.04 (0.79, 1.38)	0.76		
Yellow fever vaccination?			–	–
(Yes vs No)	1.03 (0.63, 1.69)	0.91		

HR > 1 implies a higher probability of being confirmed ZIKV RNA negative in saliva (lower likelihood of ZIKV persistence); HR < 1 implies a lower probability of being confirmed ZIKV RNA negative in saliva (increased likelihood of ZIKV persistence).

*ZIKV* Zika virus, *BMI* body mass index, *HR* hazard ratio, *CI* confidence interval.

#### Sweat

After adjusting for BMI and yellow fever vaccination, viral clearance in sweat was 1.34 times (95% CI 0.95–1.91; p = 0.09) more likely to be ZIKV RNA negative among patients living in a household with three or more members compared to households with less than three members, with marginal statistical significance (Table 5).

**Table 5:** Univariate and multivariable interval censored proportional hazards model regression estimates of relative risk of being confirmed negative for ZIKV in sweat.

Selected baseline factors	Univariate model		Multivariable model	
	Univariate HR (95% CI)	Chi-sq p value	Adjusted HR (95% CI)	Chi-sq p value
Sex				
Male	1.00			
Female	1.25 (0.94, 1.66)	0.13		
Age group			–	–
≤ 35	1.00			
> 35	0.87 (0.67, 1.13)	0.29		
Education level			–	–
Secondary or lower	1.00			
Post-secondary	1.24 (0.92, 1.66)	0.16		
BMI category (kg/m^2^)				
Normal weight (18.5–24.99)	1.00		1.00	
Overweight or obese (≥ 25.0)	0.79 (0.60, 1.06)	0.12	0.77 (0.53, 1.12)	0.16
Household members (excluding self)				
< 3	1.00		1.00	
≥ 3	1.16 (0.87, 1.54)	0.32	1.34 (0.95, 1.91)	0.09
Fever today or past 30 days			–	–
(Yes vs No)	0.85 (0.60, 1.22)	0.38		
Rash today or past 30 days			–	–
(Yes vs No)	1.02 (0.31, 3.39)	0.98		
Pruritus today or past 30 days			–	–
(Yes vs No)	1.32 (0.77, 2.27)	0.32		
Non purulent conjunctivitis today or past 30 days			–	–
(Yes vs No)	0.87 (0.665, 1.17)	0.35		
Joint pain today or past 30 days			–	–
(Yes vs No)	0.88 (0.63, 1.24)	0.47		
Periarticular edema today or past 30 days			–	–
(Yes vs No)	0.95 (0.72, 1.26)	0.72		
Yellow fever vaccination?			–	–
(Yes vs No)	1.41 (0.87, 2.31)	0.16	1.49 (0.87, 2.57)	0.14

HR > 1 implies a higher probability of being confirmed ZIKV RNA negative in sweat (lower likelihood of ZIKV persistence); HR < 1 implies a lower probability of being confirmed ZIKV RNA negative in sweat (increased likelihood of ZIKV persistence).

*ZIKV* Zika virus, *BMI* body mass index, *HR* hazard ratio, *CI* confidence interval.

#### Rectal swabs

Participants aged 35 years or older were 1.37 times (95% CI 1.01–1.87; p = 0.0054) more likely to clear ZIKV RNA in rectal swabs than their younger counterparts if both groups reported being vaccinated against yellow fever (Table 6).

**Table 6:** Univariate and multivariable interval censored proportional hazards model regression estimates of relative risk of being confirmed negative for ZIKV in rectal swabs.

Selected baseline factors	Univariate model		Multivariable model	
	Univariate HR (95% CI)	Chi-sq p value	Adjusted HR (95% CI)	Chi-sq p value
Sex			–	–
Male	1.00			
Female	1.056 (0.80, 1.39)	0.70		
Age group				
≤ 35	1.00		1.00	
> 35	1.56 (1.20, 2.01)	0.0008		0.0005
Education level				
Secondary or lower	1.00		–	–
Post-secondary	0.90 (0.68, 1.18)	0.45		
BMI category (kg/m^2^)			–	–
Normal weight (18.5–24.99)	1.00			
Overweight or obese (≥ 25.0)	0.96 (0.73, 1.27)	0.79		
Household members (excluding self)			–	–
< 3	1.00			
≥ 3	0.98 (0.74, 1.29)	0.89		
Fever today or past 30 days			–	–
(Yes vs No)	1.11 (0.78, 1.58)	0.55		
Rash today or past 30 days			–	–
(Yes vs No)	2.12 (0.67, 6.68)	0.20		
Pruritus today or past 30 days			–	–
(Yes vs No)	1.07 (0.63, 1.84)	0.80		
Non purulent conjunctivitis today or past 30 days			–	–
(Yes vs No)	1.11 (0.83, 1.47)	0.49		
Joint pain today or past 30 days			–	–
(Yes vs No)	0.98 (0.70, 1.35)	0.88		
Periarticular edema today or past 30 days			–	–
(Yes vs No)	1.07 (0.81, 1.41)	0.63		
Yellow fever vaccination?				
(Yes vs No)	1.20 (0.73, 1.99)	0.47		0.09
Interaction (Yellow fever vaccination*Age group)				0.0054
Stratified for yellow fever vaccinated = Yes				
Age > 35 (vs age ≤ 35)	1.37 (1.01, 1.87)			
Stratified for yellow fever vaccinated = No				
Age > 35 (vs age ≤ 35)	6.46 (2.25, 18.5)			
Stratified for age group ≤ 35				
Vaccinated for yellow fever (vs not vaxed)	1.83 (0.90, 3.71)			
Stratified for age group > 35				
Vaccinated for yellow fever (vs not vaxed)	0.39 (0.17, 0.89)			

HR > 1 implies a higher probability of being confirmed ZIKV RNA negative in rectal swabs (lower likelihood of ZIKV persistence); HR < 1 implies a lower probability of being confirmed ZIKV RNA negative in rectal swabs (increased likelihood of ZIKV persistence).

*ZIKV* Zika virus, *BMI* body mass index, *HR* hazard ratio, *CI* confidence interval.

#### Vaginal secretions

Non-purulent conjunctivitis and BMI were significantly associated with ZIKV RNA persistence in vaginal secretions. Women with non-purulent conjunctivitis were 1.46 times (95% CI 1.02–2.10; p = 0.04) more likely to clear the virus than women who did not report this symptom. Moreover, being overweight or obese was associated with a 33% (95% CI 5–52%; p = 0.026) lower rate of ZIKV RNA clearance compared to normal-weighted women (Table 7).

**Table 7:** Univariate and multivariable interval censored proportional hazards model regression estimates of relative risk of being confirmed negative for ZIKV in vaginal secretions.

Selected baseline factors	Univariate model		Multivariable model	
	Univariate HR (95% CI)	Chi-sq p value	Adjusted HR (95% CI)	Chi-sq p value
Age group			–	–
≤ 35	1.00			
> 35	1.01 (0.74, 1.36)	0.97		
Education level			–	–
Secondary or lower	1.00			
Post-secondary	1.05 (0.76, 1.45)	0.78		
BMI category (kg/m^2^)				
Normal weight (18.5–24.99)	1.00		1.00	
Overweight or obese (≥ 25.0)	0.75 (0.54, 1.05)	0.09	0.67 (0.48, 0.95)	0.026
Household members (excluding self)			–	–
< 3	1.00			
≥ 3	0.87 (0.63, 1.21)	0.40		
Fever today or past 30 days			–	–
(Yes vs No)	0.85 (0.58, 1.24)	0.39		
Rash today or past 30 days*			–	–
(Yes vs No)	1.79 (0.24, 13.4)	0.57		
Pruritus today or past 30 days			–	–
(Yes vs No)	0.71 (0.34, 1.49)	0.37		
Non purulent conjunctivitis today or past 30 days				
(Yes vs No)	1.29 (0.92, 1.80)	0.14	1.46 (1.02, 2.10)	0.04
Joint pain today or past 30 days			–	–
(Yes vs No)	1.38 (0.90, 2.10)	0.14		
Periarticular edema today or past 30 days			–	–
(Yes vs No)	0.80 (0.55, 1.16)	0.24		
Yellow fever vaccination?			–	–
(Yes vs No)	1.02 (0.57, 1.82)	0.96		

HR > 1 implies a higher probability of being confirmed ZIKV RNA negative in vaginal secretions (lower likelihood of ZIKV persistence); HR < 1 implies a lower probability of being confirmed ZIKV RNA negative in vaginal secretions (increased likelihood of ZIKV persistence).

*Excluding 1 participant without rash.

*ZIKV* Zika virus, *BMI* body mass index, *HR* hazard ratio, *CI* confidence interval.

#### Semen

Age, joint pain, and history of yellow fever vaccination were factors associated with ZIKV RNA persistence in semen. Patients older than 35 years and presence of joint pain showed a lower rate of ZIKV RNA clearance compared to younger patients [66% (95% CI 22–85%; p = 0.013)] and the absence of this symptom [60% (95% CI 1–84%; p = 0.047)]. On the other hand, yellow fever vaccination had the opposite effect, with vaccinated participants being 4.09 times (95% CI 1.05–16.0; p = 0.043) more likely to have an rRT-PCR negative result for ZIKV in semen than their unvaccinated counterparts (Table 8).

**Table 8:** Univariate and multivariable interval censored proportional hazards model regression estimates of relative risk of being confirmed negative for ZIKV in semen.

Selected baseline factors	Univariate model		Multivariable model	
	Univariate HR (95% CI)	Chi-sq p value	Adjusted HR (95% CI)	Chi-sq p value
Age group			–	–
≤ 35	1.00		1.00	
> 35	0.59 (0.36, 0.95)	0.032	0.34 (0.15, 0.78)	0.013
Education level			–	–
Secondary or lower	1.00			
Post-secondary	1.11 (0.63, 1.96)	0.73		
BMI category (kg/m^2^)			–	–
Normal weight (18.5–24.99)	1.00			
Overweight or obese (≥ 25.0)	0.86 (0.49, 1.51)	0.60		
Household members (excluding self)				
< 3	1.00		1.00	
≥ 3	0.73 (0.44, 1.22)	0.22	0.60 (0.29, 1.23)	0.16
Fever today or past 30 days			–	–
(Yes vs No)	0.72 (0.31, 1.68)	0.45		
Rash today or past 30 days			–	–
(Yes vs No)	0.99 (0.24, 4.11)	0.99		
Pruritus today or past 30 days			–	–
(Yes vs No)	0.92 (0.40, 2.16)	0.86		
Non purulent conjunctivitis today or past 30 days			–	–
(Yes vs No)	0.93 (0.54, 1.59)	0.78		
Joint pain today or past 30 days				
(Yes vs No)	0.60 (0.34, 1.03)	0.06	0.40 (0.16, 0.99)	0.047
Periarticular edema today or past 30 days			–	–
(Yes vs No)	1.07 (0.67, 1.71)	0.77		
Yellow fever vaccination?				
(Yes vs No)	0.92 (0.34, 2.47)	0.87	4.09 (1.05, 16.0)	0.043

HR > 1 implies a higher probability of being confirmed ZIKV RNA negative in semen (lower likelihood of ZIKV persistence); HR < 1 implies a lower probability of being confirmed ZIKV RNA negative in semen (increased likelihood of ZIKV persistence).

*ZIKV* Zika virus, *BMI* body mass index, *HR* hazard ratio, *CI* confidence interval.

The role of the following covariates was not examined in the model due to the very low or absence of incident events: HIV (n = 1), syphilis (n = 2), HBV (n = 0), HCV (n = 1), dengue (n = 0), and chikungunya (n = 0).

## Discussion

In this 1-year follow up cohort study, we investigated the detection and persistence of ZIKV RNA in various body fluid specimens from 260 ZIKV-infected participants. Our study also aimed to identify potential risk factors for ZIKV persistence and explore the possibility of recurrent ZIKV infection within 1 year of the initial acute infection. We observed that ZIKV RNA was present in all body fluid specimens, with varying duration and frequency of detection.

Previous studies have indicated that ZIKV viremia is a transient phenomenon, with higher positive detection rates occurring during the acute phase of the disease^12,23–25^. We found that the frequency of ZIKV RNA detection in plasma was the lowest (35%) compared with the other specimens analyzed, with the 95th percentile of time until the loss of detection of 17 and 28 days for males and females, respectively. This finding contrasts with previously reported results of 41 days^12^. Inconsistent results could be attributed to several factors, such as the type of specimen used (plasma versus serum), different protocols for laboratory diagnosis, and variations in the characteristics of the study population. Recent research has highlighted the importance of selecting the appropriate blood fraction for optimal detection of ZIKV RNA, with some studies indicating that specific fractions may offer higher sensitivity than others^26–28^. Another explanation for the relatively short persistence of ZIKV RNA in plasma was the high median rRT-PCR Ct values (33.2; IQR 31.0–35.7), suggestive of lower viral loads observed in the plasma specimens. ZIKV RNA persistence in whole blood has critical implications for blood donation and diagnostic purposes since this is the most employed specimen for ZIKV laboratory detection^25^.

In the multivariable analysis, educational level was an independent risk factor for ZIKV delayed clearance in plasma. Participants with post-secondary education had a 28% lower virus clearance rate than those with secondary or lower education. The association of educational level and clearance of a body fluid in a virus disease was reported among Ebola virus (EBOV) disease survivors in Sierra Leone, where education level and living with extended family were significantly associated with the prolonged virus persistence in semen, suggesting a potential sociodemographic link^29^. This link, however, has no plausible explanation yet.

Our study corroborates previous findings showing that urine is a more sensitive specimen with a longer detection window than plasma^30–32^. Almost all male (97.4%) and female (93.5%) participants tested positive for ZIKV RNA in this specimen with a lengthy detection for both sexes (72 and 92 days in male and female participants, respectively). In addition to the fact that urine collection does not require specialized personnel or laboratory facilities, this non-invasive specimen collection can increase access to testing and may positively impact the acceptability of asymptomatic contact individuals.

Sex and age were significant factors associated with ZIKV persistence in urine. Participants over 35 years of age had a 24% lower virus clearance rate, and female participants cleared the virus 47% faster than male participants. These findings are consistent with previous infectious disease research, particularly in patients infected with SARS-CoV-2^33–35^. The age-related impairment of the immune system and differences in immune system activation between sexes may contribute to explaining the different rates of viral clearance^36^.

The presence of ZIKV RNA in saliva has been largely documented^9,10,12,21,25,37,38^. In our cohort, 69.7% of the males and 64.1% of the females presented at least one positive test in this fluid. These findings align with those of Musso et al.^9^, who reported a 57.1% detection rate and differed from another investigation in which only 4.8% of participants had detectable ZIKV RNA^12^. Our study revealed that ZIKV RNA could persist in saliva specimens for an extended period, with a maximum detection of 84 days in males and 63 days in females following symptom onset. These findings support using saliva as another alternative and less invasive specimen for ZIKV diagnosis, especially within the first week of symptom onset.

Our findings support the presence of ZIKV RNA in the excretory system of naturally infected patients, as observed for hepatitis C virus (HCV) detection in eccrine sweat glands of chronically infected patients^39^. We previously reported simultaneous detection of ZIKV RNA in sweat and other body fluids^16^. In the current study, we further demonstrated that ZIKV RNA was detectable in 30% of the study participants. The longest detection of ZIKV RNA in sweat was 58 days in male and 65 days in female participants, which is longer than the 40 days observed in an Ebola patient^40^. In addition, isolated RNA detection in sweat was observed in several patients after total clearance in other body fluids collected during the same study visit. This would eliminate the possibility of cross-contamination during specimen collection. However, the impact of these findings and the role of sweat as a transmission route are still to be further investigated.

In a previous publication, we reported the detection of ZIKV RNA in rectal swabs, suggesting that direct contact with infected mucosa could be a potential transmission route^13^. Although not the first to report detection in rectal fluid, our study is the first longitudinal study to assess the presence of the virus in this fluid over 12 months. In the present manuscript, we had the opportunity further to investigate this from a larger cohort of participants. We found that ZIKV RNA was detected in rectal swabs of more than half of the male participants (56.6%) and two-thirds of the female participants (66.3%), with similar detection times after the onset of symptoms (78 and 82 days for males and females, respectively). The literature on this subject is scarce. In a case report by Li et al., ZIKV RNA was detected in a stool specimen 3 days after the onset of fever in a naturally infected patient^41^. The virus clearance rate from rectal swabs was 37% higher among participants above 35 years of age and vaccinated against yellow fever compared to their younger vaccinated counterparts. A possible explanation is the cross-reactivity with antibodies from the yellow fever vaccine, facilitating faster clearance of ZIKV. An animal model has shown that the yellow fever vaccination protects against ZIKV infection^42^. We also observed a trend for lower ZIKV viral loads in vaccinated participants, as indicated by the median Ct values in rectal swabs (31.8 among vaccinated participants and 30.8 in unvaccinated participants; IQR 29.7–34.5 and 28.8–32.4, respectively). Regarding antibody cross-reactivity, it is worth noting that multiple flaviviruses, including the four serotypes of DENV, are prevalent in the region where our study was conducted^43^. Therefore, further tests with preexisting immunity to DENV are necessary to better understand ZIKV-specific responses^44,45^.

Experimentally, ZIKV was able to infect mice^41^ and adult macaques^46^ through the anorectal mucosa, leading to detectable viremia with subsequent testicular damage and congenital defects in the offspring of pregnant mice^33^. In humans, however, the amount of infectious viral particles needed to allow transmission through this route remains unknown. A case of anal sex transmission of ZIKV was reported between a man with recent travel to an area of active ZIKV transmission and his non-traveling male partner^47^. Also, using ZIKV RNA persistence in semen/vaginal fluids to approximate infectiousness duration, the risk of male-to-male transmission had the highest estimated probability [1.3% (95% CI 0.4–6.0%)] per anal sex act than male-to-female per vaginal/anal sex act [0.4% (95% CI 0.3–0.6%)] or female-to-male transmission per vaginal sex act [0.1% (95% CI 0–0.8%)]^2^.

ZIKV RNA was present in the vaginal fluids of 60.7% of the 184 participants in our study, a higher frequency when compared to a study conducted in Puerto Rico, where detection was observed in only 1.7% of 119 women^12^. We also found longer detection at 67 days after symptom onset than in previous studies^12,32,48–53^.

We raised the possibility of vaginal secretion contamination with menstrual blood. However, after analysis of paired visits from the same participant, we found that among the positive vaginal secretion specimens, only 14.4% showed simultaneous detection with blood. Consequently, the contamination was unlikely to occur. A similar finding was reported by Prisant et al. showing evidence of viral shedding in cervical mucus on day 11 after the onset of symptoms, despite virus clearance from the patient's blood and urine samples^54^. Our observation suggests that viral replication may occur in the lower female genital tract, as supported by other studies reporting the presence of culture confirmed ZIKV in vaginal secretions^55^. Moreover, the evidence of female-to-male sexual transmission^56^ and experimental studies^57,58^ support a possible viral replication in the female genital tract.

BMI ≥ 25 kg/m^2^ was an independent risk factor for delayed ZIKV clearance in vaginal secretions. This association aligns with similar findings in other diseases^59^. For instance, during the COVID-19 pandemic, being overweight or obese was associated with prolonged respiratory tract viral shedding^60,61^. One possible explanation is that chronic inflammation and impairment of the immune system response, related to the induced insulin resistance in obesity, could hinder viral clearance^59^. After adjusting for BMI, we found that the viral clearance rate was 46% higher in women who reported non-purulent conjunctivitis. This finding is noteworthy and warrants further investigation.

We also detected ZIKV in breast milk up to 2 weeks after symptom onset, aligning with some studies included in a systematic review^62^. The strength of our study, however, lies in the serial collection and length of maternal follow-up. Nonetheless, the risk factors associated with viral persistence in this specimen could not be assessed due to the limited number of breastfeeding women enrolled. It is noteworthy that none of the infants were infected.

Various studies have established the presence of ZIKV RNA in semen specimens^3,63^. Our study detected ZIKV RNA in the semen of 69.3% of men with symptomatic infection with a 95th percentile of 113 days, and the maximum detection was 174 days. Similar results were reported by Paz Bailey et al., with a 95th percentile of 120 days and a maximum window of RNA detection of 191 days^12^. Moreover, several studies have identified long-term shedders for up to several months^19,28,64–71^. Of note, the participant with ZIKV RNA detection 174 days after the onset of symptoms had another detection at 265 days with two negative results in the 3-month interval between these two collection dates. The Ct value of the 265-day specimen was 29.58, indicating a high viral load. This raises the question of possible ongoing ZIKV replication through a reservoir located in the reproductive tract. Previous studies on human kinetics and animal models have suggested that the testis, epididymis, prostate, and seminal vesicles could play a role in the persistence of ZIKV infection^72^. Moreover, long-term detection and persistence of ZIKV in semen have been associated with inflammation of the male genital tract^17^.

Amongst all body fluids, semen has shown the lowest Ct values up to 30 days post-onset of symptoms, indicating the presence of higher viral loads in the male reproductive tract. Older age and joint pain were independent risk factors for delayed ZIKV clearance in semen, whereas yellow fever vaccination was associated with faster clearance. Men older than 40 years were more likely to test positive for ZIKV RNA in semen than men aged 40 years or younger, similar to those reported in a study of EBOV in Liberia^73^. Additionally, a study in Sierra Leone also revealed a dose–response relationship with age. Men aged ≤ 25 years were 3.17 times more likely to test negative for EBOV RNA in semen, and men aged 26–35 years exhibited a 1.85 times higher likelihood of testing negative compared to men aged > 35 years^29^. The association of joint pain as a predictor of delayed viral clearance may be explained by an exacerbated inflammatory response and the ability of ZIKV to evade the host immune response, leading to viral persistence and enhancing viral pathogenesis^74^.

Although most infections occur through a mosquito bite, ZIKV can also be transmitted sexually. The World Health Organization issued its guidelines on the prevention of sexual transmission in which it recommends the use of condoms for 3 months in cases where the infection occurred in men and 2 months when women were infected^3^. Long cohort studies were not available at the time of the guideline's publication. Our study has identified that 8.1% (3.3–15.7) of the male participants still had the persistence of ZIKV RNA in semen after 3 months of symptom onset. This does not necessarily indicate infectiousness but raises the issue of whether this is the ideal period for condom use or whether it should be increased. This has particular importance when applied to couples who are planning to conceive.

Recurrent ZIKV rRT-PCR detection, as per our study’s definition, was observed in 30% of the cohort after viral clearance. Although suggestive, we could not classify these participants as having a ZIKV reinfection or a ZIKV reactivation event. Further investigations, such as immunological, RNA sequencing, and infectivity studies, are necessary to determine the nature of these detections and their implications for public health (i.e., the possibility of reactivation or reinfection events with the potential for transmission, including sexual and vertical transmission, even in asymptomatic cases).

Our study has several strengths: (1) our study features a 1-year follow-up of ZIKV participants, involving the serial collection of samples from various body fluids; (2) remarkably, our cohort exhibited excellent follow-up rates, with fewer than 15% of patients lost to follow-up; (3) additionally, we conducted longitudinal testing of all samples for dengue and chikungunya infections, enabling us to exclude the influence of these co-infections as variables associated with ZIKV persistence; (4) lastly, our study allowed us to observe multiple instances of recurrent detection of ZIKV RNA in participants several months after the initial infection, suggesting a need for further investigation of these events.

Our study has limitations: (1) we did not use whole blood for ZIKV rRT-PCR detection, which could have improved ZIKV RNA detection, especially in asymptomatic household contacts with possible low viral loads; (2) although the study detected ZIKV RNA presence in all the specimens analyzed and also evaluated the factors associated with the persistence of the virus, the presence of infectious viral particles (virus isolation) was not performed. Therefore, we cannot determine the infective capacity of each specimen over time since the mere presence of viral RNA in a specimen does not imply active replication and infectivity; (3) our findings were based mainly on symptomatic participants and may not represent ZIKV dynamics from asymptomatic infected patients; (4) Finally, our findings may not be fully representative of the Brazilian population of ZIKV-infected patients. This limitation arises from the fact that, among the three selected initially Brazilian sites spanning the north, northeast, and southeast regions, most participants were enrolled from the north site. This discrepancy can be attributed to the lower number of ZIKV cases in the two other regions when the study received final ethical approval to commence. This situation resulted from the study being established amid a ZIKV outbreak.

## Conclusions

We found that ZIKV RNA was detectable for extended periods in urine, sweat, rectal swabs, and semen. Urine was the most frequent and persistently positive specimen, especially during the early acute phase of the disease, compared to plasma specimens. Therefore, we recommend using alternative specimens for ZIKV laboratory diagnosis to improve the probability of detecting ZIKV RNA throughout the infection. We also demonstrated that the longest detection duration was in semen, supporting previous research demonstrating prolonged ZIKV RNA presence with high viral loads in semen specimens.

Given the association between a history of yellow fever vaccination and the virus clearance rate in certain body fluids, further research is warranted.

Our study emphasizes the importance of continued monitoring and follow-up of individuals infected with ZIKV and the need for effective prevention measures to reduce the risk of transmission. In addition, the findings provide valuable information about the persistence and insight into the potential recurrence of ZIKV infection, highlighting the need for ongoing surveillance and research.

## Methods

### Study design

We designed a prospective, longitudinal cohort study of symptomatic patients with ZIKV-positive real-time reverse transcriptase-polymerase chain reaction (rRT-PCR) tests in the blood (plasma) and/or urine specimens, as well as their symptomatic or asymptomatic household/sexual contacts collected during the screening visit. Participants were men and women aged 18 years and above, with acute illness presenting with rash, recruited from two Brazilian sites (Manaus-Northern and Recife-Northeastern Brazil) between July 2017 and June 2019.

The participants in the study underwent a comprehensive data collection process. The enrollment questionnaire gathered sociodemographic information, including biological sex, race/ethnicity, formal education, marital status, and vital signs. A study physician evaluated participants, collecting extensive baseline clinical information that included comorbidities, a history of arbovirus infections (DENV, ZIKV, CHIKV, and yellow fever), immunization history, clinical manifestations, results for exams requested or referred to specialists, physical examination, other clinical or laboratory diagnoses, exam results, and referrals to specialists.

The participants were followed up for 12 months after enrollment informed consent, and body fluid specimens (blood, semen, vaginal/menstrual secretions, saliva, sweat, urine, rectal swab, and breast milk, if applicable) were tested for ZIKV RNA by rRT-PCR at 2, 4, 10, 20, 30, 60, 90, 120, 150, 180, 210, 240, 270, 300, 330, and 360 days following the recruitment visit. HIV, syphilis, pregnancy, HBV, and HCV tests were conducted at baseline, six-, and 12-months post-enrollment.

The study protocol and detailed baseline characteristics of the cohort have been published elsewhere^22,75^.

### Zika virus detection

Vaginal, rectal, and sweat swab samples were diluted in 1 mL of sterile Hank’s Balanced Salt Solution (ThermoFisher Scientific, https://www.thermofisher.com). Semen, urine, and saliva were collected in sterile containers. All samples were refrigerated and transported to the laboratory within 2 h. Upon arrival, the samples were transferred to pre-identified cryotubes, adhering to specified numbers and volumes for defined aliquots. These aliquots were stored in appropriate cryoboxes in an Ultrafreezer at − 86 °C until processing. ZIKV detection was carried out using rRT-PCR, processing 200 μL of each specimen for RNA extraction through an automated nucleic acid purification platform, utilizing the Maxwell 16 Viral Total Nucleic Acid Purification Kit (Promega Corporation, https://www.promega.com), in a final volume of 70 μL. The same protocol was applied to RNA extraction across all body fluids and research centers.

ZIKV detection was performed by rRT-PCR employing a commercial kit, namely ZDC (for Zika, Dengue, and Chikungunya). The kit was approved by The Brazilian Health Regulatory Agency (ANVISA), registry #80142170032 (https://www.bio.fiocruz.br). The validation of the ZIKV test for body fluids other than plasma and urine was carried out under the guidance of a WHO expert. Samples were deemed positive when the target amplification was detected within 38 amplification cycles, coupled with the positive detection of an internal control reaction. This control reaction involved an RNA virus-like particle that was individually added to each specimen before the RNA extraction process. The test results were categorized as positive/detected, negative/undetected, or indeterminate/not interpreted. Positive or negative specimens were considered valid results, whereas indeterminate/non-interpretable results were excluded from the analysis.

### Outcome and variables

The flowchart of study participants (S1) depicts the screening and enrollment figures at each study site, disaggregated by participant sex and type (index case or household/sexual contact). The baseline characteristics, including sociodemographic factors and vital signs at enrollment, were stratified by sex and reported using descriptive statistics. Categorical variables were presented using frequencies and percentages, while normally distributed quantitative variables were summarized using means and standard errors. Medians, interquartile ranges, and minimum and maximum values were reported for skewed, non-normal quantitative variables.

We defined virus clearance as the first occurrence of two consecutive rRT-PCR negative results, at least 30 days apart, following the preceding positive result. This interval was measured in days.

The study investigated the potential relationship between viral persistence and host, sociodemographic factors, and the presence of symptoms. The following variables were selected for exploration: sex, age group, educational level, Body Mass Index (BMI), number of household members (excluding self), presence of symptoms such as fever, rash, pruritus, non-purulent conjunctivitis, joint pain, periarticular edema, and yellow fever vaccination status.

### Survival analysis

To estimate the rate of ZIKV persistence in body fluids, a non-parametric Kaplan–Meier (KM) interval-censored (IC) survival analysis was used^76^. The endpoint for persistence was defined as the interval of time from the earliest onset of ZIKV symptoms at the screening visit (V0) to the first of two consecutive negative results in each analyzed specimen. For asymptomatic cases, the date of the screening visit that yielded a positive ZIKV test for urine and/or blood was used. Censoring intervals were considered left-censored if the participants had no ZIKV positive result on a particular specimen, right-censored in the absence of a confirmed negative ZIKV result during the study follow-up period, and interval-censored if the event of interest (confirmed negative ZIKV result) occurred between the date of a ZIKV positive and negative result of a particular body fluid specimen during the study follow-up period.

In case of fluctuation of rRT-PCR results between ZIKV positive and ZIKV negative from V0, the event date was the date of the first of the two consecutive ZIKV negatives occurring later than 30 days from V0, as long as the interval between the event date and the date of the immediately following ZIKV positive result was more than 60 days. This ZIKV positive occurring > 60 days after the event date was considered recurrent detection.

The interval-censored KM ZIKV persistence curves were plotted, stratified by sex, for each specimen separately. A proportional hazards (PH) interval-censored (IC) semi-parametric model was used to adjust for potential confounders. Variables whose difference in − 2LogL (Log-likelihood) univariate PH-IC model estimate from the null model had a p value < 0.20 were fitted together in a multivariable model. Variables were retained in the model if their omission from the multivariable model resulted in a significant change in the − 2LogL, i.e., p value < 0.2 level. Variables with univariate − 2LogL p value above 0.20 were added back in the multivariable model and were retained if significant at p < 20 level. Interactions between covariates in the PH IC model were studied for possible inclusion in the final multivariable model if they were highly significant (p < 0.01). Two-sided tests and 5% significance levels were used, and 95% confidence intervals (CIs) were reported for all relevant parameters. The SAS statistical package was used for the statistical analyses^77^, and R Version 3.3.3 for the plotting graphics.

### Ethical approval

Ethical permission was granted from the World Health Organization Ethics Review Committee (WHO ERC), Protocol ID: ERC.0002786; the Brazilian National Research Ethics Commission (CONEP) (CAAE: 62518016.6.1001.0008); the Institutional Ethics and Research Committee of the Evandro Chagas National Institute of Infectious Diseases, Fiocruz, Rio de Janeiro (CAAE: 62518016.6.2002.5262); the Institutional Ethics and Research Committee of the Aggeu Magalhães Research Center, Fiocruz, Recife (CAAE: 62518016.6.2001.5190) and the Institutional Ethics and Research Committee of the Tropical Medicine Foundation, Manaus, Amazonas (CAAE: 62518016.6.2003.0005). All methods were performed in accordance with the Brazilian regulations (Resolution 441 of May 12th, 2011, from the Brazilian National Health Council).

### Supplementary Information

Supplementary Information.

## Data Availability

The datasets used and analyzed during the current study will be available on reasonable request from corresponding author.
