# Supplementary material for: Detection and persistence of Zika virus in body fluids and associated factors: a prospective cohort study
Source: Sci Rep. 2023 Dec 6;13:21557. doi: 10.1038/s41598-023-48493-8 (PMC10700488; doi:10.1038/s41598-023-48493-8)
Supplement: Supplementary file 1 — Supplementary Information. [file 41598_2023_48493_MOESM1_ESM.pdf]

Detection and persistence of Zika virus in body fluids and associated factors: A prospective cohort study

Guilherme Amaral Calvet, Edna Oliveira Kara, Camila Helena Aguiar Bôtto-Menezes, Marcia da Costa Castilho, Rafael Freitas de Oliveira Franca, Ndema Habib, Armando Menezes Neto, Gerson Fernando Mendes Pereira, Silvana Pereira Giozza, Ximena Pamela Díaz Bermúdez, Tatiana Jorge Fernandes, Kayvon Modjarrad, Patrícia Brasil, Nathalie Jeanne Nicole Broutet, and Ana Maria Bispo de Filippis on behalf of ZIKABRA Study Team

Supplementary Fig. 1: Participants flowchart

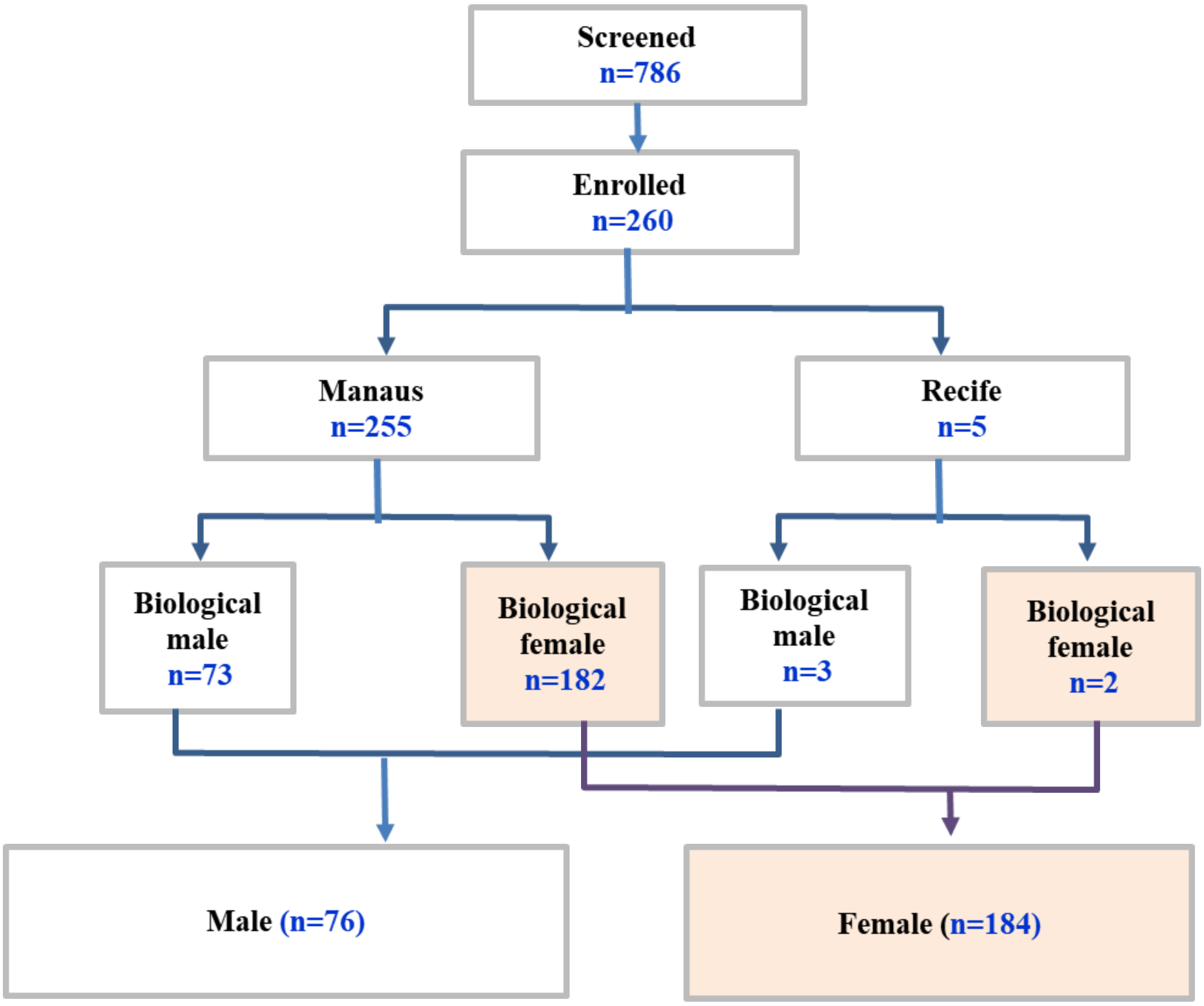

Supplementary Table 1. Number of samples collected and rRT-PCR results by body fluid in males (n=76) and females (n=184) during the study

Body Fluid	Number of positive ZIKV rRT-PCR results		Number of negative ZIKV rRT-PCR results		Number of not interpreted ZIKV rRT-PCR results		Total samples
	Male	Female	Male	Female	Male	Female	
Plasma	61	130	1176	2991	-	1	4359
Urine	312	634	925	2490	-	-	4361
Saliva	91	169	1068	2765	3	9	4105
Sweat	32	62	1117	2855	3	8	4077
Rectal	74	200	1068	2730	15	6	4093
Semen*	132	-	839	-	26	-	997
Vaginal Secretions	-	174	-	2756	-	8	2938
Right Breast Milk**	-	7	-	52	-	-	59
Left Breast Milk**	-	4	-	56	-	-	60

* One participant did not provide semen

** Six breastfeeding women

Supplementary Table 2. First incidence of detectable ZIKV in body fluid

**Days from symptom onset**	**Cumulative hazard (%) (95%CI)**								
	**Saliva**		**Rectal fluid**		**Sweat**		**Vaginal secretions**	**Breastmilk**	**Semen**
	**Male**	**Female**	**Male**	**Female**	**Male**	**Female**	**Female**	**Female**	**Male**
**2**	1.4 (0.2, 9.6)	5.7 (3.0, 10.7)	0.0	4.3 (2.1, 8.7)	0.0	0.0	5.0 (2.5, 9.7)	16.7 (2.5, 72.7)	0.0
**3**	15.5 (8.9, 26.2)	19.0 (13.7, 26.0)	8.6 (3.9, 18.1)	12.2 (8.0, 18.3)	0.0	0.0	16.3 (11.4, 22.9)	50.0 (19.6, 88.9)	5.6 (2.2, 14.3)
**4**	33.8 (24.1, 46.1)	34.2 (27.4, 42.1)	24.5 (16.0, 36.5)	30.5 (24.1, 38.2)	1.5 (0.2, 10.4)	0.7 (0.1, 4.6)	32.0 (25.4, 39.8)	50.0 (19.6, 88.9)	14.1 (7.8, 24.6)
**5**	49.3 (38.4, 61.4)	50.0 (42.5, 58.0)	36.1 (26.1, 48.6)	43.3 (36.1, 51.2)	7.7 (3.3, 17.5)	3.4 (1.4, 7.9)	45.2 (37.9, 53.3)	50.0 (19.6, 88.9)	21.1 (13.3, 32.6)
**6**	59.2 (48.1, 70.6)	62.7 (55.2, 70.2)	43.4 (32.7, 55.9)	53.2 (45.7, 61.0)	9.2 (4.3, 19.4)	6.1 (3.2, 11.3)	52.2 (44.7, 60.2)	50.0 (19.6, 88.9)	28.2 (19.2, 40.2)
**7**	64.8 (53.8, 75.6)	65.2 (57.8, 72.5)	46.3 (35.4, 58.7)	57.5 (50.0, 65.1)	10.8 (5.3, 21.3)	6.7 (3.7, 12.1)	56.0 (48.5, 63.9)	50.0 (19.6, 88.9)	33.8 (24.1, 46.1)
**10**	70.4 (59.6, 80.5)	70.9 (63.7, 77.8)	57.9 (46.7, 69.6)	63.0 (55.7, 70.4)	18.5 (10.9, 30.2)	11.5 (7.3, 17.8)	63.7 (56.2, 71.1)	66.7 (32.4, 95.4)	43.7 (33.1, 56.0)
**14**	70.4 (59.6, 80.5)	71.5 (64.4, 78.3)	59.4 (48.1, 70.9)	64.3 (56.9, 71.5)	23.1 (14.6, 35.3)	13.5 (8.9, 20.1)	65.0 (57.5, 72.3)	66.7 (32.4, 95.4)	53.5 (42.5, 65.4)

CI, confidence interval

Supplementary Fig. 2: rRT-PCR cycle thresholds in body fluids following symptoms onset

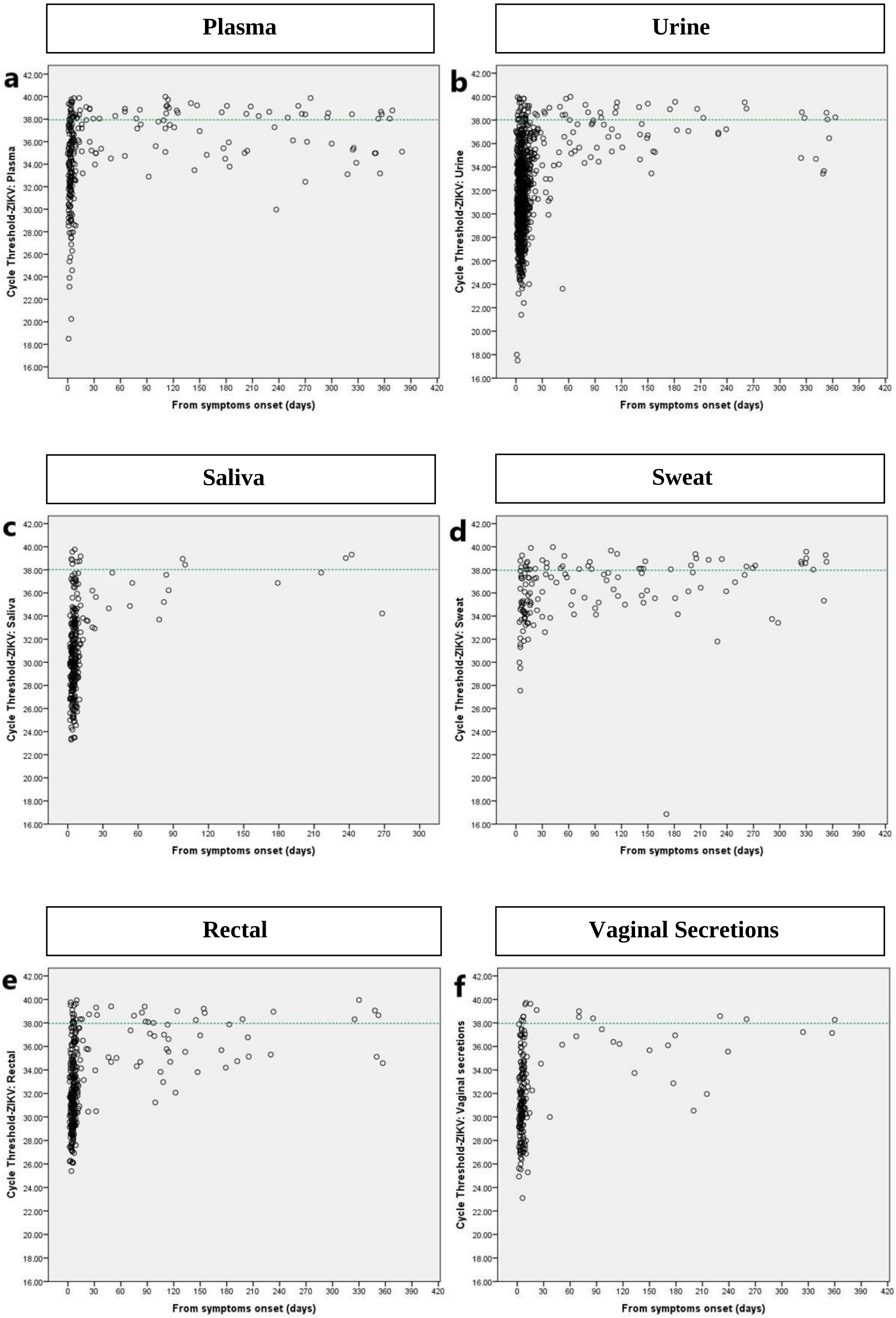

Semen

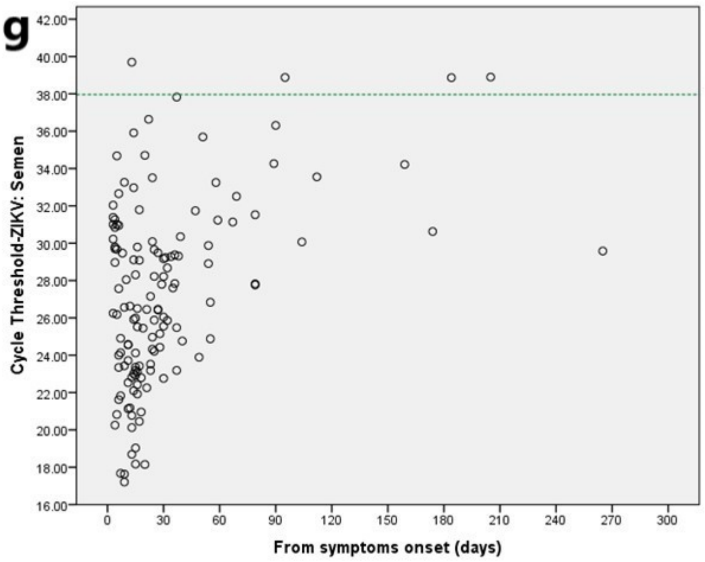

Distribution of Cycle threshold (Ct) values by body fluid. Each “dot” represents a ZIKV rRT-PCR result. The dashed line represents the detection limit of the assay. a. Plasma, b. Urine, c. Saliva, d. Sweat, e. Rectal, f. Vaginal secretions, g. Semen.

ZIKABRA Study Team

Guilherme Amaral Calvet[1], Patrícia Brasil[1], Tatiana Jorge Fernandes[1], Edna Oliveira Kara[2], Ndema Habib[2], Nathalie Jeanne Nicole Broutet[2], Camila Helena Aguiar Bôtto-Menezes[3,4], Marcia da Costa Castilho[3], Marcus Vinicius Guimarães de Lacerda[3], Rafael Freitas de Oliveira Franca[5], Armando Menezes Neto[5], Morganna Costa Lima[5], Gerson Fernando Mendes Pereira[6], Silvana Pereira Giozza[6], Maria Cristina Pimenta de Oliveira[6], Ximena Pamela Díaz Bermúdez[7], Kayvon Modjarrad[8], Lydie Trautman[8], Ana Maria Bispo de Filippis[9], André Luiz de Abreu[10], and Carlos Alexandre Antunes de Brito[11]

1. Acute Febrile Illnesses Laboratory, Evandro Chagas National Institute of Infectious Diseases, Oswaldo Cruz Foundation, Rio de Janeiro, Rio de Janeiro, Brazil.
2. Department of Sexual and Reproductive Health and Research, World Health Organization, Geneva, Switzerland.
3. Department of Malaria, Tropical Medicine Foundation Doctor Heitor Vieira Dourado (FMT-HVD), Manaus, Amazonas, Brazil.
4. School of Health Sciences, Amazonas State University (UEA), Manaus, Amazonas, Brazil
5. Department of Virology and Experimental Therapy, Institute Aggeu Magalhães, Oswaldo Cruz Foundation, Recife, Pernambuco, Brazil
6. Department of HIV/AIDS, Tuberculosis, Viral Hepatitis and Sexually Transmitted Infections (DATHI), Ministry of Health, Brazil

7. Department of Public Health, University of Brasilia, Brasília, Brazil.
8. Emerging Infectious Diseases Branch, Walter Reed Army Institute of Research, Silver Spring, MD, United States of America.
9. Flavivirus Laboratory, Oswaldo Cruz Institute, Oswaldo Cruz Foundation, Rio de Janeiro, Rio de Janeiro, Brazil
10. General Coordination of Public Health Laboratories (CGLAB/DAEVS/SVS/MS), Brasília-DF, Brazil

11. Clinical Hospital of Federal University, Department of Internal Medicine, Recife, Pernambuco, Brazil
